# The Medical Association of Central New York

**Published:** 1903-12

**Authors:** C. A. Greenleaf

**Affiliations:** Rochester, N. Y.


### SOCIETY PROCEEDINGS.

### The Medical Association of Central New York.

*Thirty-sixth Annual Meeting, held at Auburn, N. Y., September 22, 1903.*

Reported by C. A. GREENLEAF, M. D., Rochester, N. Y.

THE meeting was called to order at 10.30 a. m., by Dr. John L. Heffron, of Syracuse, president.

The first order of business is the reading of the minutes of the last meeting.

Dr. C. A. Greenleaf, Secretary: Heretofore this report has been printed in the Transactions and it has been customary in the past to omit it, and for that reason I move that the secretary's report be omitted.

Motion seconded and carried.

The president then announced the standing committees. Committee on Credentials, Dr. A. H. Brown, Auburn; Dr. G. G. Lewis, Syracuse, and Dr. Frank Hoxie, of Union Springs. Business Committee: Dr. David M. Totman, Syracuse; Dr. William S. Cheeseman, Auburn, and Edward J. Wynkoop, of Syracuse. Reception Committee: Dr. Joseph F. Creveling, Dr. John Gerin, of Auburn, and Dr. William W. Skinner, of Geneva, and from the Auburn Medical Society, Dr. Sheldon Voorhees, Dr. M. P. Conway, and Dr. S. E. Austin. Publication Committee: Dr. Wm. C. Krauss, of Buffalo; the secretary, Clarence A. Greenleaf, of Rochester, and the treasurer, Dr. William M. Brown, of Rochester.

The treasurer's report was then read.

The Treasurer: I would like to say that in 1900 the treasurer was directed to collect the dues each year from every member, whether he attended the meetings or not. The first year that was not observed, and when I assumed the office I had a great deal of trouble in finding a list of the members of the society. I went back to the old secretary's books and culled out a list as well as possible, but my acquaintance was limited, and since then I have been sending bills to dead people and people who have moved to other parts of the world, and if there are mistakes sometimes in rendering bills I want the members to be lenient with me. I try to collect all the dues that are owing to the society, and I wish that there was some way of getting a more accurate list of the members. There has been no record kept of the deaths, and I do not know who are members and who are not members. If anyone present has any suggestion to make of any way by which we can get a more complete list of the members, the treasurer will be very glad to hear from him.

The President: What will you do with the report of the treasurer?

It was moved and seconded that the report of the treasurer be adopted. Carried.

The President: The question which the treasurer brought up I think is a very vital one. I happen to hold in my hand the only copy which is known to be in existence of the Constitution and By-laws, and this was printed in 1883. It seems to me that the time is already here when we ought to have a new copy of the By-laws and Constitution, and with them also a list of the membership of the society. A great many changes I understand have been made in the Constitution since this was printed, and I think at the proper time that would be a subject for action on the part of the society.

Dr. J. P. Creveling: I move that a committee be appointed to revise the By-laws and Constitution, if necessary, and to make a new list of the members.

Motion seconded.

The President: I would like very much if that committee would be nominated by the Association. I am not sufficiently acquainted with the members of the Association who would be active on such a committee.

Dr. Creveling: Mr. President, I would suggest Dr. John O. Roe, of Rochester; Dr. D. M. Totman, of Syracuse, and the secretary, Dr. C. A. Greenleaf, of Rochester. Motion seconded and carried.

President Heffron read his address, entitled, Our Relations to a Few of the Problems in State Medicine.

Dr. Charles E. Congdon, of Buffalo, then read a paper entitled, Personal experiences in puerperal infection.

Dr. G. Griffin Lewis, of Syracuse, read a paper entitled, Keratoconus.

Dr. Lucien Howe, of Buffalo, read a paper entitled, Examinations of the eye which the general practitioner can and should make.

Dr. D. H. Murray, of Syracuse, read a paper entitled, Recto-colonic lavage, with description of new and improved tubes.

Dr. William S. Cheeseman read a paper entitled, Gastro-enterostomy for pyloric obstruction.

The Association adjourned for luncheon, served by the Cayuga County Medical Society.

AFTERNOON SESSION.

The president, Dr. HEFFRON, called the meeting to order in the rooms of the Knights of Columbus, at 2 o'clock.

Dr. William L. Wallace, Syracuse, read a paper entitled, Cystitis in the Female.

Dr. William B. Jones, Rochester, read a paper entitled, The Bladder Troubles of Old Men.

Dr. Nathan Jacobson, Syracuse, read a paper entitled, Contribution to the Surgery of Prostatic Hypertrophy.

Dr. Charles G. Stockton, Buffalo, read a paper entitled, Cholecystitis.

Dr. B. Farquhar Curtis, New York, read a paper entitled, Notes on the Surgery of the Gall-bladder and Ducts.

Dr. F. W. Higgins, Cortland, read a paper entitled, A Plea for the Conservative Treatment of Appendicitis.

Dr. E. S. Van Duyn, Syracuse, read a paper entitled, The Influence of the Leucocyte Count in the Decision for or Against Immediate Operation.

Dr. H. R. Hopkins, Buffalo, read a paper entitled, Malignant Endocarditis of the Right Heart.

Dr. A. L. Beahan, Canandaigua, read a paper entitled, Uterine Myoma.

Dr. Thomas H. Halstead, ————, read a paper entitled, Cerebral Complications of Acute Mastoiditis.

Dr. B. C. Loveland, Syracuse, read a paper entitled, Fat in its Relation to Nervous Disease.

The election of officers for the ensuing year resulted as follows: president, C. A. Vander Beek, of Rochester; 1st vice-president, Charles G. Stockton, of Buffalo; 2d vice-president, A. H. Brown, of Auburn; secretary, C. A. Greenleaf, of Rochester; treasurer, William M. Brown, of Rochester.

The secretary then read the names of those eligible for permanent membership.

*Onondaga County.*—M. M. Lucid, E. S. Van Duyn, S. E. Crane, W. Coe McKeeby, J. C. Shroudy, A. G. Doust, Nathan A. Munroe, Theresa Bannan, Frederick Flaherty, H. A. MacGruer, L. A. Saxer, J. H. Kerand.

*Cayuga County.*—Ledra Heazlit, W. H. Coe, Jr.

*Ontario County.*—C. C. Lytle, W. W. Skinner.

Dr. J. Gerin: I move that the recommendation of the committee be adopted and the several persons received as members of the Association. Carried.

The president declared such members duly elected.

Dr. H. R. Hopkins: Before adjourning it gives me great pleasure, on behalf of the guests and of the Medical Association of Central New York, in view of the exceedingly delightful entertainment they have received in attendance upon this meeting, to move a vote of thanks to the Medical Society of Cayuga County, and to the Knights of Columbus, for their hospitality in entertaining us. Carried.

Dr. Vander Beek: Under Article 18 of the Constitution this Association may at each annual meeting elect two honorary members. Dr. B. F. Curtis and Dr. J. Riddle Goffe, of New York, have honored us by their presence and have instructed us with their work. I therefore move that these two gentlemen be elected to honorary membership in this Association. Carried

The President: The meeting is then declared adjourned to meet for the dinner at the Osburne House. The hour is appointed for 7 o'clock sharp.

